# Standardized Pomegranate (Pomella^®^) and Red Maple (Maplifa^®^) Extracts and Their Phenolics Protect Type I Collagen by the Inhibition of Matrix Metalloproteinases, Collagenase, and Collagen Cross-Linking

**DOI:** 10.3390/molecules27227919

**Published:** 2022-11-16

**Authors:** Huifang Li, Tithi Roy, Samuel T. Boateng, Hao He, Chang Liu, Weixi Liu, Dongli Li, Panpan Wu, Navindra P. Seeram, Jean Christopher Chamcheu, Hang Ma

**Affiliations:** 1 School of Biotechnology and Health Sciences, Wuyi University, Jiangmen 529020, China; 2 International Healthcare Innovation Institute (Jiangmen), Jiangmen 529020, China; 3 Bioactive Botanical Research Laboratory, Department of Biomedical and Pharmaceutical Sciences, College of Pharmacy, University of Rhode Island, Kingston, RI 02881, USA; 4 School of Basic Pharmaceutical and Toxicological Sciences, College of Pharmacy, University of Louisiana at Monroe, Monroe, LA 71209, USA

**Keywords:** pomegranate (*Punica granatum*), red maple (*Acer rubrum*), punicalagin, ginnalin A, collagenase, cross-linking, anti-skin-aging

## Abstract

Phenolics enriched pomegranate fruit (Pomella^®^) and red maple leaf (Maplifa^®^) extracts and their major phenolic constituents have demonstrated beneficial skin effects through the protection of human skin keratinocytes from oxidative-stress-induced damage. However, their mechanisms of protection of cutaneous collagen are still unclear. Herein, the collagen protective effects of Pomella^®^ and Maplifa^®^, and their major bioactive phytochemicals, namely, punicalagin (PA) and ginnalin A (GA), respectively, were evaluated using enzymatic assays including collagenase, anti-glycation and cell-based models as well as computational methods. The importance of the modulatory effects was validated at the protein level for type I collagen and matrix metalloproteinases (MMPs) using human-skin-derived keratinocytes. The synergistic collagenase inhibitory effects upon combinations of Pomella^®^ + Maplifa^®^ and PA + GA at a combination ratio of 1:2 and 1:1, respectively, were evaluated using their combination index (CI; a well-established assessment of synergism). Pomella^®^ (50–400 µg/mL), Maplifa^®^ (100–800 µg/mL), PA (50–400 µM), and GA (50–400 µM) dose-dependently inhibited collagenase activity by 26.3–86.3%, 25.7–94.0%, 26.2–94.0%, and 12.0–98.0%, respectively. The CI of the anti-collagenase activity of Pomella^®^ and Maplifa^®^ ranged from 0.53–0.90, while that of PA and GA (12.5/12.5 and 25/25 µM) ranged from 0.66 and 0.69, respectively, suggesting a synergistic inhibitory effect. Interestingly, in the cell-based assays by Western blotting, Pomella^®^ and Maplifa^®^ reduced the protein expression levels of collagen degradation enzymes (MMPs), while simultaneously increasing that of type I collagen in epidermoid carcinoma A431 cells. This is the first report to show that these extracts exert synergistic collagen protective effects. Taken together, these findings provide molecular insights into the usefulness of Pomella^®^ and Maplifa^®^ or their phenolics as bioactive ingredients for skin care products to slow down aging and enhance skin tone.

## 1. Introduction

In humans and other animals, collagen is the most abundant protein and constitutes the major component of the extracellular matrix (ECM) of the dermal component of the skin, which accounts for three-quarters of the dry weight of the skin [1]. Because of its critical position between the cutaneous basement membrane zone and the dermal component of the skin, collagen has a pivotal role in the maintenance of the physical barrier and structural integrity of the skin [1]. Increased degradation, damage, and absence of collagen is known to lead to declined levels of the ECM, which are characteristic biomarkers related to skin-aging conditions such as the formation of skin wrinkles or denuded skin in hereditary dermatological conditions such as dystrophic epidermolysis bullosa and pseudoxanthoma elasticum [2,3]. The process of collagen degradation is often modulated by a number of biochemical factors including non-enzymatic reactions such as the formation of advanced glycation end-products; AGEs [4] and enzymatic peptide cleavage reactions catalyzed by various matrix metalloproteinases (MMPs) as well as collagenases [5]. The dysregulated expression of collagenase is linked to several skin-aging processes, such as the formation of skin wrinkles, which is a consequence of collagen depletion, and a weakened dermal ground matrix [6]. Therefore, the identification of inhibitors of collagenase or MMPs could represent potential management strategies for combating aging-related skin conditions such as skin wrinkle formation. Published studies support that the anti-wrinkle effects of some extracts from medicinal plants and functional foods may contribute to their ability to inhibit matrix-protein-degradation-related enzymes including collagenase and elastase [7,8]. Considering the role of ECM proteins in skin integrity, downregulating degradation enzymes such as MMPs is important to maintaining skin homeostasis both for cosmetic and anti-skin-aging purposes.

A pomegranate fruit extract was reported to reduce the formation of skin wrinkle via the inhibition of MMP activity in an ultraviolet (UV) B-induced skin photoaging hairless mice model [9]. A chemically standardized pomegranate fruit extract Pomella^®^ was shown to have anti-photoaging effects in UVA- and UVB-treated SKU-1064 human skin fibroblasts in vitro [10]. Additionally, further study revealed that phenolic-enriched pomegranate products improved UVB-induced damage in a reconstituted human skin model [11]. Similar protective effects of a phenolic extract of the Amur maple (*Acer ginnala*) species were also reported against UVB-induced cytotoxicity in human dermal fibroblasts by modulating several cellular signaling pathways including MAPK/AP-1, NF-κB, and TGFβ/Smad [12]. Phenolics in the extracts of maple species, namely, ginnalins, are modulators of the neurogenic locus notch homolog protein 1 (NOTCH1) that maintain the homeostasis of the cellular metabolism of human epidermal keratinocytes [13]. Moreover, we recently reported that both Pomella^®^ and Maplifa^®^ (a proprietary phenolic-enriched red maple leaf extract) exhibit skin protective effects by alleviating oxidative-stress-induced toxicity in human keratinocytes [14,15]. However, the inhibitory effects of Pomella^®^ and Maplifa^®^ on skin wrinkle formation and the related enzymes, such as collagenase and MMPs, remain undefined.

In this study, we assess the protective effects of Pomella^®^ and Maplifa^®^ along with their major phytochemicals/phenolics on skin collagen (type I) through the evaluation of their modulatory effects on collagenase, AGE-induced cross-linking, and the expression of MMPs and type I collagen. Moreover, the synergistic effects of Pomella^®^ and Maplifa^®^ on the basis of their combination indexes (CIs), providing insights into their collagen protective effects, were evaluated as compared to their individual activity. Furthermore, the major phenolic compounds of Pomella^®^ (punicalagin; PA) and Maplifa^®^ (ginnalin A; GA) were used as chemical probes to explore the mechanisms of action of the extracts. We report for the first time that Pomella^®^ and Maplifa^®^ exert synergistic inhibitory effects on collagenase activity and MMP protein expression and promote type I collagen protein expression.

## 2. Results

### 2.1. Pomella^®^ and Maplifa^®^ Inhibit Collagenase Activity and Their Combination Exerts Synergistic Effects

**Figure 1:**
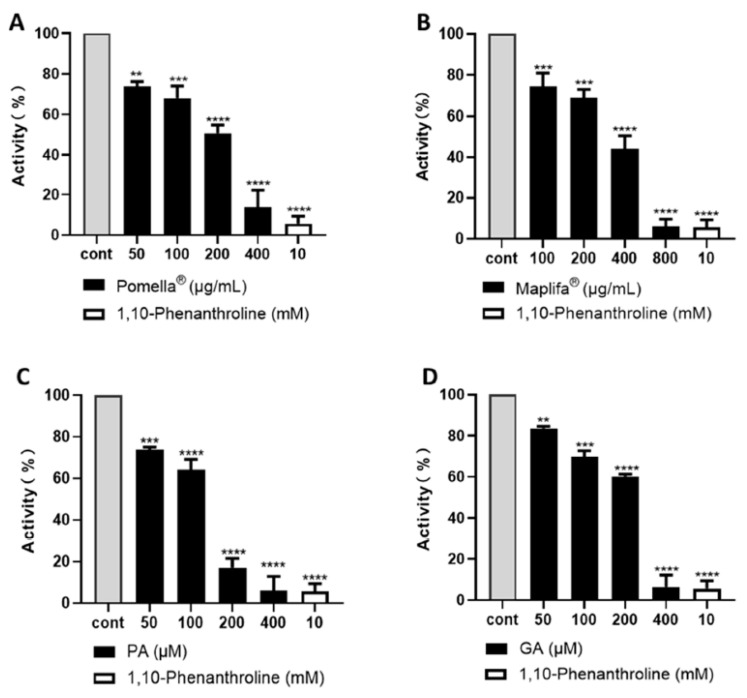
Inhibition of collagenase enzyme activity by (**A**) Pomella^®^ and (**B**) Maplifa^®^ (both at 100, 200, 400, and 800 µg/mL), and (**C**) PA and (**D**) GA (both at 50, 100, 200, and 400 µM). The inhibitory effects were determined by a colorimetric assay. Data are expressed as means ± standard deviation (S.D.) from three replicated experiments. Statistically significant difference was considered ** *p* < 0.01, *** *p* < 0.001 and **** *p* < 0.0001 when compared to the control group.

The Pomella^®^ or Maplifa^®^ treatments resulted in a concentration-dependent inhibition of collagenase activity by 26.3%, 32.0%, 49.3%, and 86.3%, and by 25.7%, 31.0%, 56.0%, and 94.0%, respectively (Figure 1A,B). The major phytochemical constituents of these extracts, punicalagin (PA) and ginnalin A (GA), also exerted a dose-dependent collagenase inhibitory activity by 26.2%, 35.9%, 83.0%, and 94.0%, and by 16.5%, 30.3%, 39.9%, and 93.7%, respectively (Figure 1C,D). The positive control 1,10-Phenanthroline (at 10 mM) was less active as compared to PA and GA and showed an inhibitory rate of 94.4%. The data from the anti-collagenase assay suggest that Pomella^®^ and Maplifa^®^ are promising collagenase inhibitors.

**Figure 2:**
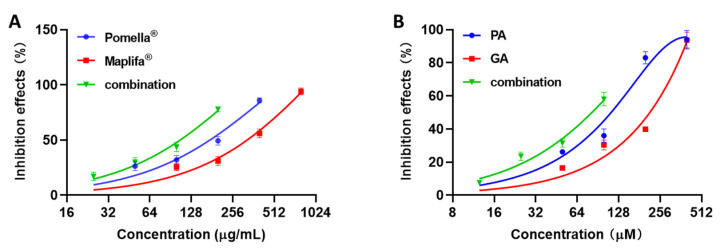
Synergistic inhibitory effects of the combinations of (**A**) Pomella^®^ + Maplifa^®^ (at a ratio of 1:2 combinations; 2.5 + 25, 25 + 50, 50 + 100, and 100 + 200 µg/mL; presented by the green lines) and (**B**) PA + GA (in a ratio of 1:1 combination; 12.5 + 12.5, 25 + 25, 50 + 50, and 100 + 100 µM; presented by the green lines), and their activity was compared to the activity of their individual sample (Pomella^®^, presented by the blue line; Maplifa^®^, presented by the red line; PA, presented by the blue line; and GA presented by the red line).

Furthermore, through the CI values of the inhibitory effects from the combination of different concentrations of both Pomella^®^ and Maplifa^®^, as well as PA and GA, we assessed the synergistic potential and observed that the combinations of Pomella^®^ and Maplifa^®^ exerted synergistic inhibitory effects on collagenase activity. The inhibitory effects of the combinations were separately obtained and compared to the activity of their individual samples (Figure 2).

Additionally, a synergistic effect was observed when PA and GA were tested at lower concentrations (12.5/12.5 and 25/25 µM), with an observed inhibitory rate of 7.5% and 23.5% and a CI of 0.87 and 0.84 (Table 1). The combinations of Pomella^®^ and Maplifa^®^ had more promising synergistic inhibitory effects on collagenase than their pure compounds, with the Pomella^®^ + Maplifa^®^ inhibitory rates being 17.3%, 29.7%, 43.7%, and 77.7%, with CI values of 0.53, 0.67, 0.90, and 0.70, respectively. 

### 2.2. Punicalagin (PA) and Ginnalin A (GA) Interact with Collagenase Protein

**Figure 3:**
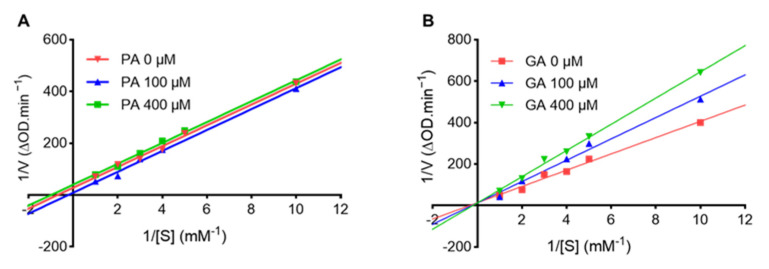
Lineweaver–Burk plots of FALGPA hydrolysis by collagenase in the presence of PA (**A**) and GA (**B**). Michaelis–Menten parameters for collagenase hydrolysis of FALGPA in the presence of PA or GA at concentrations of 100 µM and 400 µM.

To elucidate the mechanisms of the anti-collagenase activity of PA and GA, a kinetic assay was performed to determine the type of enzyme inhibition. The Lineweaver–Burk plots showed the linear relationship between 1/v and 1/[s], and the lines were in parallel or intersected at the *Y*-axis in the presence of PA or GA, respectively (Figure 3A,B), which suggested that PA is an uncompetitive inhibitor and GA is a competitive inhibitor.

This was supported by their Michaelis–Menten parameters showing a decreased *Km* and *Vmax* as the concentration of PA increased (100 to 400 µM), with an increased *Km* and maintained *Vmax* as the concentration of GA increased (100 to 400 µM) (Table 2). Therefore, our data suggest that PA and GA are different types of collagenase inhibitors, which may contribute to their synergistic inhibitory effects.

**Figure 4:**
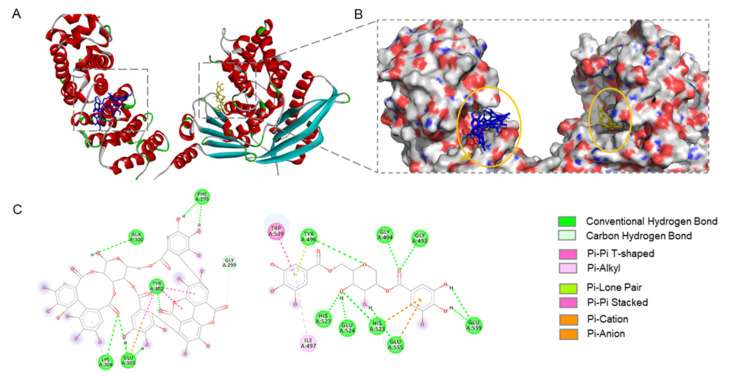
Molecular docking of the interactions between PA or GA and collagenase protein (PDB 2Y6I). Predicted binding sites of PA and GA on the two side chains of collagenase protein (**A**) and their enlarged view (**B**). Molecular forces formed between PA or GA and protein amino acid residues to stabilize the protein–ligand complex (**C**).

To further support the possible mechanisms of their synergistic effects, molecular docking was conducted to study the interactions between PA or GA and the collagenase protein. As shown in Figure 4A, PA and GA have different binding sites for the collagenase protein. PA has an estimated binding energy of −5.99 kcal/mol with an inhibition constant of 40.68 µM. It binds to the collagenase protein on the protein side chain A, which is close to the binding cavity of a collagenase ligand, tris-hydroxymethyl-aminomethane (Figure 4B) [16]. Conversely, GA fits into a binding pocket located at the opposite side chain of the collagenase protein, which is similar to the binding site of the collagenase inhibitor isoamylphosphonyl-Gly-Pro-Ala (Figure 4B) [16].

GA also has a comparable binding energy of −5.6 kcal/mol and an inhibition constant of 78.28 µM. Overall, the docking analysis revealed that GA and PA can bind to the collagenase protein at different sites, which may result in the conformational changes of the protein structure. In addition, PA and GA can form various molecular forces when they bind to the collagenase protein, which facilitate the formation of the protein–ligand complex as shown in the 3D interaction diagram (Figure 4C). For instance, the major type of molecular forces in the PA–collagenase complex are hydrogen bonds formed between the hydroxyl groups of PA and the collagenase protein’s amino acid residues including Ala300, Phe295, Tyr302, Glu303, and Lys304. Similarly, GA also forms hydrogen bonds with the collagenase protein between its hydroxyl groups and amino acid residues (His527, Glu524, His523, Glu555, and Gly559). In addition, noncovalent interactions including π–π stack, π–alkyl, and π–anion are formed between GA and the collagenase protein. 

### 2.3. Pomella^®^, Maplifa^®^, PA and GA Reduce Glycation-Induced Collagen Cross-Linking

**Figure 5:**
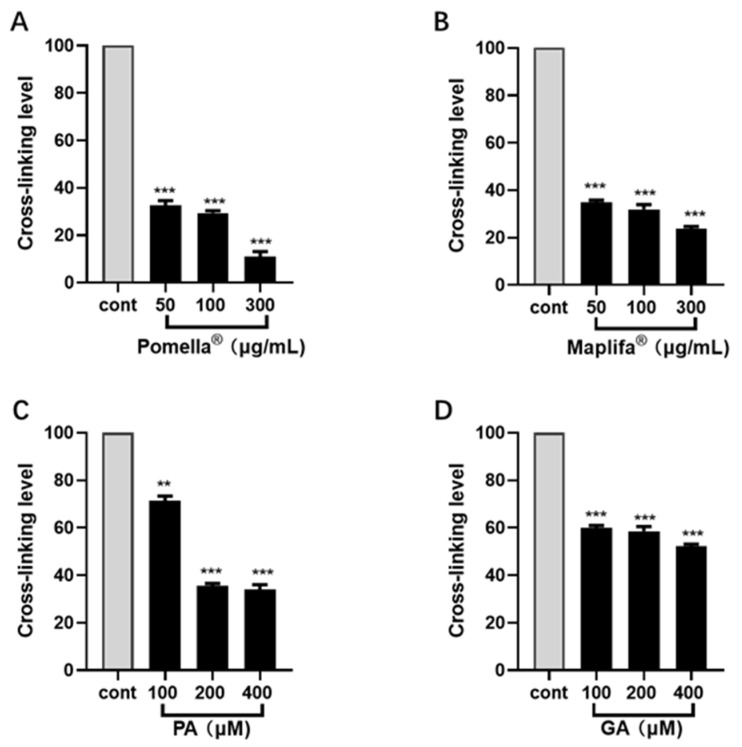
Effects of Pomella^®^ and Maplifa^®^ and their respective major phytochemical constituents, namely, punicalagin (PA) and ginnalin A (GA), on the formation of AGEs in a model of type I collagen cross-linking using a protein solution (PureCol^®^, Carlsbad, CA, USA) containing c.a. 97% type I collagen. The inhibitory effects of Pomella^®^ and Maplifa^®^ (both at 50, 100 and 300 µg/mL; (**A**,**B**), respectively) as well as PA and GA (both at 100, 200, and 400 µM; (**C**,**D**), respectively) on the levels of collagen protein cross-linking were determined by a fluorescent assay. Values are expressed as means ± standard deviation (S.D.) from three experimental replicates. Significance was defined as ** *p* < 0.01 and *** *p* < 0.001 when compared to the control group.

To further evaluate the protective effects on the collagen protein, a non-enzymatic assay was used to assess the inhibitory effects of Pomella^®^, Maplifa^®^, PA, and GA against glycation-induced collagen cross-linking. Pomella^®^ and Maplifa^®^ (at 50, 100, and 300 μg/mL) inhibited collagen cross-linking by 67.3–88.9% and 65.2–76.3%, respectively (Figure 5A,B). PA and GA (at 100, 200, and 400 μM) also significantly reduced the collagen cross-linking level by 28.6–66.0% and 40–47.9%, respectively (Figure 5C,D).

### 2.4. Pomella^®^, Maplifa^®^, PA and GA Modulated Cell Viability in Cultured Normal Human Immortalized Keratinocytes (N/TERT1) and Epidermoid Carcinoma (A431) Cell Lines at Higher Concentrations

To evaluate the cellular effects of Pomella^®^ and Maplifa^®^ and their respective phenolics (PA and GA), as well as their combinations, we first assessed their cytotoxicity in two immortalized human epidermal keratinocyte cell lines, namely, normal keratinocytes (N/TERT1) and epidermoid carcinoma (A431) keratinocytes, and selected the test doses that were not cytotoxic in both cell lines. As shown in Table 3, the cell viability of NTERT1 and A431 cells was determined, and the IC_50_ values of the extracts (Pomella^®^ and Maplifa^®^) and their respective phenolics (PA and GA) provided the basis for the selection of the concentrations used further. By Western blot assay we assessed the protein expression levels of the ECM by remodeling protein-related markers (i.e., MMPs and type I collagen). The IC_50_ values of the extracts and pure compound were higher for the normal keratinocytes (NTERT1) than the epidermoid carcinoma cells (A431). The dose-dependent cell viability data are shown in Supplementary Materials: Figure S1.

### 2.5. Pomella^®^, Maplifa^®^, PA and GA Reduced the Protein Expression of MMPs in Cultured N/TERT1 and A431 Cell Lines

**Figure 6:**
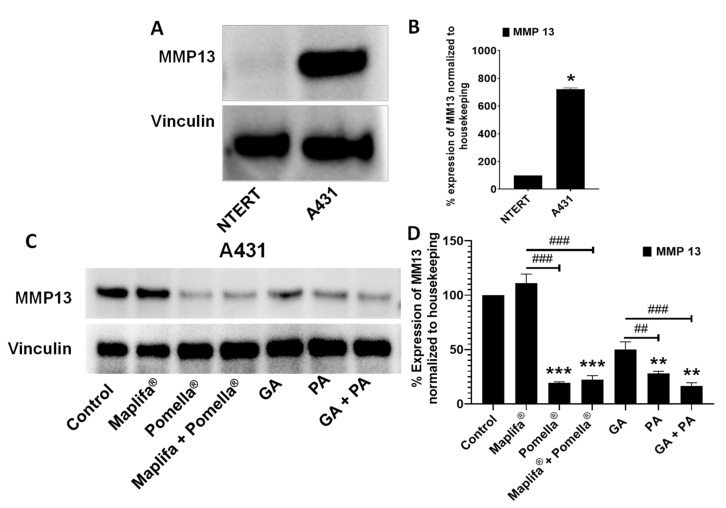
Protein expression levels of metalloproteinase 13 (MMP-13) on NTERT and A431 whole cell lysates without any treatment (**A**) and significant increase of MMP-13 expression in A431 compared to NTERT (**B**). Measurement of MMP13 expression on A431 upon treatment with Pomella^®^ and Maplifa^®^ extracts (both at 12.5 µg/mL), their pure compounds PA and GA (both at 12.5 µM) and their 1:1 combination each at 12.5 µM. The inhibitory effects were determined by the Western blot assay using A431 cell lysates (**C**). Data are expressed as means ± standard deviation (S.D.) from three independent experiments performed in duplicate (**B**,**D**). Statistically significant difference was considered * *p* < 0.05, ** *p* < 0.01, *** *p* < 0.001 when compared to the control group and ^##^ *p* < 0.01, ^###^ *p* < 0.001 when compared between individual treatment groups.

To quantify the changes in the protein expression levels of MMPs associated with the skin ECM, we first established the basal expression level of MMP-13 in both N/TERT1 and A431 cells. At the basal condition we observed that A431 cells showed significantly higher MMP-13 protein expression levels as compared to the N/TERT1 cells (72.2% vs. 10.0%, respectively (Figure 6A,B). Henceforth, the A431 cells were selected as a cellular model for further evaluations of the test samples’ effect on the MMP-13 protein expression. Compared to the control group, treatment with Pomella^®^ at a non-cytotoxic concentration (12.5 µg/mL, minimum concentrations used in the CI enzymatic assays) resulted in a decreased MMP-13 protein expression level, whilst Maplifa^®^ (25 µg/mL, minimum concentration employed in CI determination collagenase inhibitory assay) did not decrease the protein expression level of MMP-13 (Figure 6C,D). In addition, the combination of Pomella^®^ and Maplifa^®^ at a 1:1 ratio synergistically decreased MMP-13 expression by 77.6% as compared to the control group. The pure phenolic compounds from Pomella^®^ and Maplifa^®^, GA and PA, also showed inhibitory effects on MMP-13 expression. Treatment with GA and PA (both at 12.5 µM, doses used in CI determination experiments above) significantly inhibited the expression level of MMP-13 by 49.9% and 71.9%, respectively, as compared to the control group. Additionally, their combinations (1:1) synergistically suppressed MMP-13 protein expression by 83.4% (Figure 6C,D).

### 2.6. Pomella^®^, Maplifa^®^, PA and GA Modulate the Protein Expression Levels of Type I Collagen and MMP-9 in Cultured Human Epidermoid Carcinoma A431 Cells

**Figure 7:**
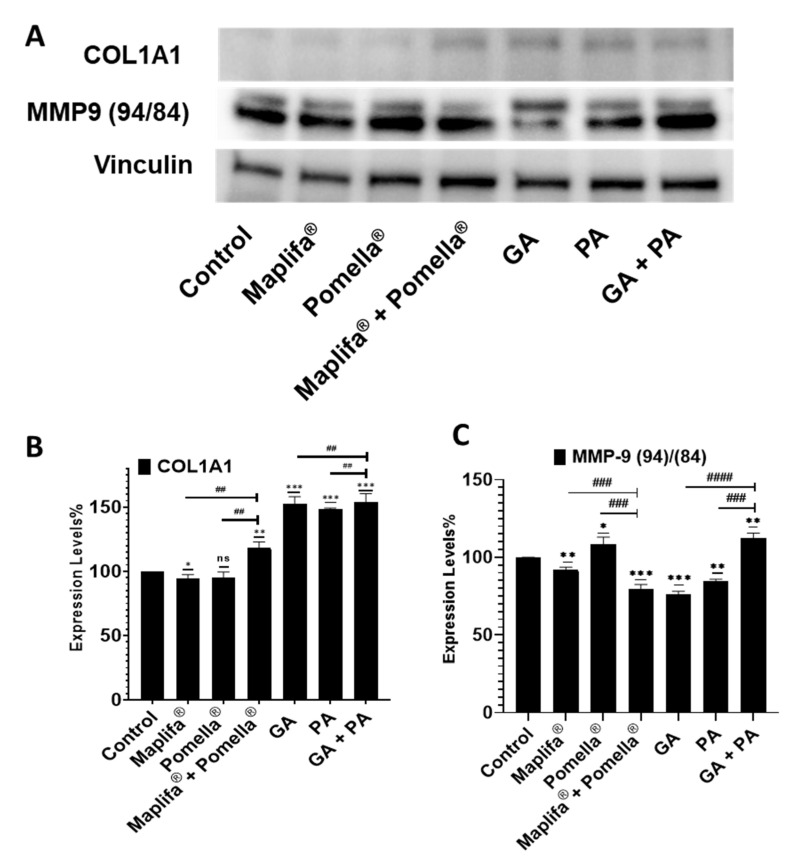
Induction of type I collagen (collagen 1A1) protein expression levels and modulation of matrix metalloproteinase 9 (MMP-9) after 48 h treatment of A431 with Maplifa^®^ (25 µg/mL), Pomella^®^ (12.5 µg/mL), Maplifa^®^ (25 µg/mL) + Pomella (12.5 µg/mL), GA (12.5 µM), PA (12.5 µM) and GA+PA (12.5 µM) by Western blotting. The inductive or inhibitory effects were analyzed by Western blot assay of A431 cell lysates (**A**). Data are expressed as means ± standard deviation (S.D.) from three independent experiments each performed in duplicate (**B**,**C** for collagen 1 A1 and MMP9 respectively). Statistically significant difference was considered, not significant (ns) compared to control, * *p* < 0.05, ** *p* < 0.01, *** *p* < 0.001 when compared to the control group and ^##^ *p* < 0.01, ^###^ *p* < 0.001, ^####^ *p* < 0.0001 when compared between individual treatment groups.

Treatment with Pomella^®^, Maplifa^®^, and their combination, as well as PA, GA, and their combination increased the protein expression levels of type I collagen (COL1A1) in A431 cells as evidenced by the observed increased protein expression levels when compared to the control group as analyzed by Western blot assay (Figure 7A, top panel; Figure 7B). 

In addition, treatment with Pomella^®^, Maplifa^®^, PA, and GA, as well as their combinations differentially modulated the protein expression level of MMP-9 (Figure 7A, bottom Panel; Figure 7C). Maplifa^®^, GA, PA and the combination of Maplifa^®^ and Pomella^®^ significantly decreased the protein expression levels of MMP-9 by 8%, 23.7%, 15.5% and 20.5%, respectively, whereas Pomella^®^ or the combination of GA and PA significantly increased MMP-9 levels as compared to the baseline control group (see Figure 7A, bottom Panel; Figure 7C). Despite the increase in MMP-9 expression by Pomella^®^, its combination with Maplifa^®^ resulted in a synergistic reduction in the MMP-9 expression level. For the pure compounds, GA and PA individually resulted in a decreased MMP-9 expression, whereas their combination did not seem to actively decrease the MMP-9 protein expression. Nonetheless, the overall observation suggests that these extracts and their purified constituents are able to downregulate MMP-9 and MMP-13 protein expression levels, suggesting a possible mechanism for the protection of the ECM in skin tissues.

## 3. Discussion

Synergism is defined as a combination of two (or more) agents exerting greater biological activity than the addition of their individual effects [17]. This is not an unusual phenomenon, particularly in the practice of traditional medicine systems, in which formulations with combined botanicals may achieve improved overall beneficial effects [17]. Therefore, it is possible that combinations of Pomella^®^ and Maplifa^®^ could exhibit a synergistic inhibition of collagenase and MMPs through different mechanisms. Notably, both of these chemically standardized extracts have been extensively studied by our group for their phytochemical constituents, leading to the identification of over seventy compounds (including ellagitannin derivatives) from Pomella^®^ [18] and over one hundred phenolics (mostly glucitol-core-containing gallotannins) from Maplifa^®^ [19]. Thus, apart from PA and GA, several other phytochemicals present in Pomella^®^ and Maplifa^®^ may also exert overall inhibitory effects on collagenase activity by binding to different sites of the enzymes to enhance their inhibitory effects. This is not surprising as similar biological effects have been observed in our previously published studies, in which Pomella^®^ and Maplifa^®^ inhibited the formation of AGEs via distinctive mechanisms, namely, by the reactive-carbonyl-species-trapping effect vs. the free-radical-scavenging effect, respectively [20,21]. Furthermore, non-enzymatic collagen cross-linking can largely contribute to the damage of the ECM integrity and in turn exacerbate the process of skin aging. Notably, our group has reported that PA exerts protective effects on type II collagen through the inhibition of protein degradation, which was shown to be associated with its binding capacity to the collagen protein [22]. This is the first report to show that Pomella^®^ and Maplifa^®^, as well as PA and GA, display inhibitory effects against cutaneous type I collagen cross-linking, which may partially contribute to the overall protective effects of collagen. To further understand these effects at the cellular level, a Western blot analysis of cultured cell lysates demonstrated a modulatory effect that was mostly associated with decreased protein expression levels of MMPs including MMPs (-9 and -13), which was corroborated by a corresponding increase in the protein expression levels of COL1A1. Despite the significant decrease in MMP-9 expression levels by GA or PA, their 1:1 combination ratio was not synergistic in decreasing MMP-9 protein expression. Therefore, further studies including evaluations of the diverse combination ratios and treatment time points are warranted to elucidate the optimal combinations of the extracts and their phenolics for their skin protective effects. More physiologically relevant conditions, such as the 3D skin-tissue-engineered models, should be further utilized to affirm the effectiveness of Pomella^®^ and Maplifa^®^ and their compounds as protective agents of skin collagen. Such studies will confirm the individual and synergistic modulatory effects on collagenase, MMP (-9 and -13) and pro-collagen type I promotion. Taken together, these observations demonstrate that these extracts and their pure phenolics significantly downregulate MMP-9 and MMP-13 protein expression levels, and suggest their positive modulatory effects on the ECM, which could protect and enhance type I collagen as well as maintain the skin integrity.

## 4. Materials and Methods

### 4.1. Chemicals, Antibodies, and Reagents

Antibodies for immunoblotting including COL1A1 (E6A8E) Rabbit mAb #39952, MMP-3 (D7F5B) Rabbit mAb #14351 and MMP-9 (D603H) XP^®^ Rabbit mAb #13667, β-Actin (13E5) Rabbit mAb #4970, horseradish-peroxidase-conjugated (HRP) anti-rabbit secondary antibodies were all obtained from Cell Signaling Technologies (Beverly, MA, USA). Pomella^®^, a commercially available and chemically standardized pomegranate fruit extract, was provided by Verdure Sciences (Noblesville, IN, USA). Our group has previously reported on the chemical standardization and preparation of Maplifa^®^, a proprietary standardized phenolic-enriched red maple leaf extract [23]. Notably, our group has extensively characterized the phytochemical constituents of both pomegranate fruit and red maple leaf extracts using liquid chromatography coupled with time-of-flight tandem mass spectrometry (LC–TOF–MS) and ultra-fast liquid chromatography coupled with electrospray ionization/time-of-flight mass spectrometry (UFLC–ESI–TOF–MS/MS), respectively, which resulted in the identification of seventy compounds (including ellagitannin derivatives) from the pomegranate fruit extract and over one hundred phenolics (mostly glucitol-core-containing gallotannins) from the red maple leaf extract [18,19]. Detailed data on the identifications and chromatograms of phytochemicals including seventy phenolics in Pomella^®^ and ninety-four phenolics in Maplifa^®^ are provided in the Supplementary Materials (Figures S2 and S3 and Tables S1 and S2). Additionally, the levels of the major phytochemicals, namely, PA (in Pomella^®^) and GA (in Maplifa^®^), were determined as 30.1 and 42.3%, respectively, by high-performance liquid chromatography (HPLC). Punicalagin (PA) and 1,10-phenanthroline were purchased from Sigma-Aldrich (St. Louis, MO, USA). Ginnalin A (GA) was isolated from Maplifa^®^ according to our previously reported method [11]. A collagenase activity kit containing enzyme protein and its substrate *N*-[3-(2-Furyl)-acryloyl]-Leu-Gly-Pro-Ala (FALGPA) was purchased from Abcam Inc. (Cambridge, MA, USA). 

Radioimmunoprecipitation assay (RIPA) buffer, Pierce Bicinchoninic acid (BCA™) protein assay kit, and the SuperSignal™ West Pico PLUS Chemiluminescent substrate detection system were purchased from ThermoFisher Scientific (Rockford, IL, USA). Mini-protean precast Tris-Glycine Gels (TGX) were from Bio-Rad (Bio-Rad Laboratories Inc., Hercules, CA, USA). Dulbecco’s modified Eagle’s medium (DMEM) was from Corning (Corning, Manassas, VA, USA). Epi-Life^®^ Growth Medium with 60 µM calcium and Cascade Biologist human keratinocyte growth supplement (HKGS) 100× (S-001–5) were from Life Technologies (Grand Island, NY, USA). The Dulbecco’s phosphate-buffered saline (DPBS), phosphate-buffered saline (PBS) 1×, defined trypsin inhibitor (DTI) 1× (R007-100), trypsin EDTA 0.25%, 1× (R25200-072), Trypsin neutralizer (TN) 1× (R-002–100), penicillin–streptomycin (Pen Strep,15140–122) (PEST) 100× were purchased from Gibco, ThermoFisher Scientific (Rockford, IL, USA), and human keratinocyte growth supplement (HKGS) 100× (S-001–5) from Cascade Biologist were purchased from VWR Corporation (Missouri City, TX, USA). USDA-approved Origin Fetal Bovine Serum (FBS) was procured from VWR Seradigm Life Science (Missouri City, TX, USA). 3-(4,5-Dimethylthiazol-2-yl)-2,5-diphenyltetrazolium bromide (MTT), dimethyl sulfoxide (DMSO) and bovine serum albumin (BSA) were purchased from Sigma–Aldrich Chemical Company (St. Louis, MO, USA) and MP Biomedicals (Irvine, CA, USA), respectively.

### 4.2. Enzyme Inhibition Kinetic Assay

The inhibitory kinetics of collagenase by PA and GA were evaluated using the previously reported method with minor modifications [24]. Briefly, a mixture of test samples (20 µL) and Tris-HCl buffer (50 µL; 50 mM; pH 7.5), and collagenase type I (10 µL; 0.5 mg/mL) was incubated in a 96-well plate at room temperature for 15 min. Next, different concentrations (0.5–5 mM) of FALGPA solution (20 µL) in Tris-HCl buffer were added to each well and the absorbance of each well was recorded at a wavelength of 355 nm using a plate reader with a kinetic mode for 30 min.

### 4.3. Cell Lines, Cell Cultures, Cytotoxicity and Viability Assay

Epidermoid carcinoma A431 cell lines were purchased from Angio-Proteomie (Boston, MA, USA) and cultured in DMEM supplemented with 5% FBS. Immortalized primary keratinocyte cell lines (N/TERT1) were generously provided by Dr. Ellen van den Bogaard (Radboud University Nijmegen, Netherlands) to J.C.C. and cultured in Epi-Life^®^ Growth Medium with 60 µM calcium with cascade Biologist human keratinocyte growth supplement (HKGS) added [25,26]. All cells were maintained in an incubator at 37 °C under an atmosphere of 95% humidity, 20% O_2_ and 5% CO_2_. The growth media of incubated cells were changed every 2–3 days until they attained 70–80% confluence, after which they were sub-cultured for experiments and/or re-passaging. DMSO was used as a vehicle to prepare a 10 mM stock solution of the test compounds. Control cells were treated with the vehicle (DMSO) at concentrations (0.01–0.2%) without affecting cell viability.

Stock solutions of the extracts (Pomella^®^ and Maplifa^®^; 100 mg/mL) and pure compounds (PA and GA; 100 mM) were prepared in DMSO and then diluted to the desired experimental concentrations with the corresponding cell growth media. Cells (65–75% of confluency) were treated with (0–100 µg/mL or µM for the extracts or pure compounds, respectively) or without the test samples in quadruplicates and incubated for 48 h. All treatment protocols and controls were prepared as previously described [27].

The MTT assay was performed to determine the cytotoxic influence of the test compounds and extracts in the cell lines used. Briefly, exponentially growing parent passage cells at a confluency of 70–85% were harvested, washed, counted using a hemocytometer, and individually seeded at a density of 2–5 × 10^3^ cells/well in a 96-well plate in their respective growth media (200 µL). Cells were incubated and monitored until reaching their logarithmic growth phase at over 70% confluence prior to the treatment. All experimental treatment concentrations were repeated in at least 8–10 wells, and the experiment was performed in 5–6 replicates. Subsequently, the cells were harvested following the MTT protocol as previously described [27] with absorbance measured at a wavelength of 562 nm using a SynergyLX, multimode microplate reader (BioTek Instruments Inc., Winooski, VT, USA). GraphPad Prism software version 9.3 (GraphPad Software, Inc., La Jolla, CA, USA) was used to calculate the various IC_50_ values as previously described [27]. 

### 4.4. Molecular Docking Analysis

Molecular docking analysis was conducted on the molecular modeling station supported by the Rhode Island IDeA Network of Biomedical Research Excellence (RI-INBRE) with an Autodock 4.2 program using the Lamarckian Genetic Algorithm (LGA). The 3D structural coordinates of PA and GA were obtained from the human metabolome database (www.HMDB.ca; accessed on 29 June 2020). The crystal structure of the collagenase protein (PDB ID: 2Y6I) was retrieved in PDB format from the RCSB protein data bank (www.rcsb.org; accessed on 29 June 2020). The collagenase protein was prepared by removing water molecules and adding force-field parameters. Known co-crystallized ligands, namely, tris-hydroxymethyl-aminomethane and isoamylphosphonyl-Gly-Pro-Ala, were used for assigning the ligand-binding domain for the docking simulations [16]. The optimized protein structure, construction of missing side chains and loops, binding site and the binding score of the ligands were obtained on the basis of their free binding energy and hydrogen bond. The 2D diagrams of the protein–ligand interactions were visualized by Discovery Studio (DS; BIOVIA Corp.; San Diego, CA, USA).

### 4.5. Collagen Cross-Linking Inhibition Assay

Glycation-induced collagen cross-linking was evaluated with the bovine type I collagen assay. A commercially available type I collagen solution that contained approximately 97% type I collagen was isolated from bovine atelocollagen (PureCol^®^, Carlsbad, CA, USA). The measurement of the level of glycated collagen was conducted in a cross-linking assay using reported methods with minor modifications [28,29]. Briefly, a reaction mixture consisting of type I collagen (Advanced BioMatrix Inc; PureCol^®^, Catalog #5005; Carlsbad, CA, USA; 1.5 mg/mL), test samples, methylglyoxal (MGO; 5 mM), and sodium azide (10 mM) in phosphate-buffered saline (PBS) was incubated at 37 °C for 30 days. Next, the level of cross-linked collagen was monitored by measuring the fluorescence intensity with excitation and emission wavelengths at 340 and 435 nm, respectively, with a plate reader (Molecular Devices, Sunnyvale, CA, USA). In addition, each sample was tested without the enzyme and served as a background reading in order to exclude the interference from the potential inherent fluorescence of the sample.

### 4.6. Preparation of Cell Lysates, Western Blot Assay and Analysis

Cell lysates were prepared after subjecting 3 × 10 cm^2^ plates seeded with about 3000 cells each and treated with individual test agents and their combinations for 48 h, following the reference protocol [27]. Briefly, harvested cells were lysed by incubating in cell lysis RIPA buffer supplemented with protease and phosphatase inhibitors; 1 mM PMSF (phenylmethylsulfonyl fluoride), 10 mM RIPA buffer, 1 mM protease inhibitor cocktail (1 mM EDTA, 20 mM NaF, 0.5% NP-40, 1% Triton X-100) and 0.5% Na_3_VO_4_ on ice. Samples were then centrifuged at 14,000 rpm for 25 min at 4 °C, and the supernatants were collected and the concentration of cleared protein lysate was quantified using the BCA protein assay kit (Thermo Scientific, Pierce Rockford, IL, USA) according to the manufacturer’s protocol. A readout was obtained using the Eppendorf BioPhotometer D30, and aliquots were also withdrawn either for immediate use, or for storage at −80 °C for later use in the Western blot assay [27].

The Western blotting assay was carried out to evaluate the effect of the test compounds on extracellular matrix (ECM) components using previously described protocols [27]. Briefly, cell lysates were denatured in 2× Laemmli sample buffer at 95 °C and then 25 µg of protein per lane for each sample were loaded onto 4–12% polyacrylamide ready mini-PROTEAN TGX gels, followed by electrophoresis at 120 V for 1 h and 20 min. The resolved proteins were transferred onto a nitrocellulose filter membrane using a Trans-Blot Turbo Transfer Pack (BioRad Laboratories; Hercules, CA, USA). The presence of proteins was indicated using a ponceau staining before proceeding to washing (2×) under tap water and TBS-T wash buffer. Unspecific epitopes in the membrane were blocked with fat-free dry milk (7%)/Tween 20 (1%) in 20 mM Tris-buffered saline (TBS or wash buffer) at room temperature on a rotating shaker for 45 min. The membranes were incubated overnight at 4 °C for 2 h in buffer containing the corresponding primary monoclonal or polyclonal antibodies (1:250 to 1:1000 dilution). Blots were then washed three times (5 min each) with PBST and incubated with anti-mouse or anti-rabbit horseradish-peroxidase (HRP)-conjugated secondary antibody (1:2000 dilution). Afterward, blots were re-washed three times (5 min each) with PBST and MilliQ-water, followed by exposure to Enhanced Chemiluminescence Plus West Pico for 1 to 5 min, and autoradiographed in a Bio-Rad Gel-Doc System (Bio-Rad Laboratories, Hercules, CA, USA). The loading consistency was verified using a house-keeping protein (β-actin) by stripping and re-probing blots with monoclonal β-actin primary antibody (Sigma, St. Louis, MO, USA) following previously described procedures [30]. The Western blot bands were visualized and imaged using the Bio-Rad ChemiDoc™ detection system. Densitometry was performed using a BioRad digitized scientific imaging analysis software system program (Quantity One). The β-actin densitometric readouts were used for normalization, linear ranges of band densities were established through multiple exposures of blot, and the analysis and data presented was based on at least two different experiments. The data were analyzed for statistical significance using one-way ANOVA as computed with the GraphPad Prism software package (version 9.3.1) [27].

### 4.7. Statistical Analyses

Data are presented as mean ± standard deviation (S.D.) of at least three replicated experiments. Two-tailed unpaired student’s t-test or one-way ANOVA with Tukey post-test was used for statistical analysis of the data using the GraphPad Prism software version 8.0. Statistical significance for all tests was considered when *p* < 0.05 (*), *p* < 0.01 (**), *p* < 0.001 (***), and *p* < 0.0001 (****).

## 5. Conclusions

In summary, Pomella^®^, Maplifa^®^, PA, and GA inhibited the activity of collagenase, which was enhanced by their various combinations, suggesting that they exerted synergistic inhibitory effects. This was further supported by data obtained from the molecular docking study. Further studies on the combinations of Pomella^®^ and Maplifa^®^, and PA and GA using a relevant 2D skin keratinocyte culture model confirmed their downregulatory potentials in the investigated MMPs (-9 and -13), which corresponded to the increased production of type I collagen. This suggested that PA and GA contributed to the overall collagen protective effects of their parent extracts Pomella^®^ and Maplifa^®^, which warrants further investigation in physiologically relevant in vivo models. Such studies as tissue-engineered skin matrices, ex vivo skin explant cultures and preclinical animal models could strongly validate the overall anti-skin-wrinkle efficacy prior to clinical translation. Nevertheless, this is the first report on the synergistic effects of Pomella^®^ and Maplifa^®^ on a skin-wrinkle-related enzyme, supporting their usefulness as bioactive ingredients for skin care, anti-skin-aging and beauty products. Indeed, Pomella and Maplifa as well as their combinations are the best candidates for further in vivo and/or clinical studies.

## Figures and Tables

**Table 1:** CI values for the combinations of Pomella^®^ + Maplifa^®^ and PA + GA on the inhibition of collagenase activity.

Pomella^®^ + Maplifa^®^ (µg/mL)	Combination Index (CI)	Synergistic Inhibition	PA + GA (µM)	Combination Index (CI)	Synergistic Inhibition ^a^
12.5 + 25	0.53	+	12.5 + 12.5	0.87	+
25 + 50	0.67	+	25 + 25	0.84	+
50 + 100	0.90	+	50 + 50	1.34	−
100 + 200	0.70	+	100 + 100	1.47	−

^a^ Synergistic effects are shown in ‘+’(CI < 1); No synergistic effects are shown in ‘−’ (CI > 1).

**Table 2:** Michaelis–Menten kinetic parameters for collagenase-catalyzed hydrolysis of FALGPA in the presence of PA and GA at concentrations of 100 and 400 µM.

Compound	Concentration (µM)	*Km* (mM)	*Vmax* (mM/min)	Inhibition Type
PA	0	7.743	0.191	Uncompetitive
	100	1.425	0.035	
	400	1.006	0.025	
GA	0	2.890	0.074	Competitive
	100	4.027	0.078	
	400	4.957	0.078	

**Table 3:** The effect of the extracts Maplifa^®^ and Pomella^®^, and their respective major compounds GA and PA in two human skin cell lines NTERT1 and A431.

Sample	IC_50_ Values	
	NTERT1	A431
Maplifa^®^ (µg/mL)	160.6 ± 11.46	99.2 ± 0.6
Pomella^®^ (µg/mL)	26.6 ± 5.27	38.8 ± 1.3
PA (µM)	46.1 ± 5.68	30.5 ± 1.0
GA (µM)	99.8 ± 3.75	49.5 ± 1.2

## Data Availability

Raw data obtained in this study are available from the corresponding authors upon reasonable request.

## Supplementary Materials

The following supporting information can be downloaded at: https://www.mdpi.com/article/10.3390/molecules27227919/s1, Figure S1: The cell viability at four (4) different concentration against A431 and NTERT1 for Maplifa^®^, Pomella^®^, GA, PA and their combinations. Figures S2 and S3: Total ion chromatograms of Pomella^®^ and Maplifa^®^ in liquid chromatography coupled with time-of-flight tandem mass spectrometry (LC-TOF-MS/MS), respectively. Table S1: Characterization of phenolic compounds in Pomella® by LC-TOF-MS/MS. Tables S2 and S3: Characterization of phenolic compounds in Pomella^®^ and Maplifa^®^ by LC-TOF-MS/MS, respectively.
