# Supplementary material for: Standardized Pomegranate (Pomella®) and Red Maple (Maplifa®) Extracts and Their Phenolics Protect Type I Collagen by the Inhibition of Matrix Metalloproteinases, Collagenase, and Collagen Cross-Linking
Source: Molecules. 2022 Nov 16;27(22):7919. doi: 10.3390/molecules27227919 (PMC9696304; doi:10.3390/molecules27227919)
Supplement: Supplementary file 1 [file molecules-27-07919-s001.zip › molecules-2008670-supplementary.pdf]

SUPPLEMENTARY MATERIALS

Standardized Pomegranate (Pomella®) and Red Maple (Maplifa®) Extracts and their Phenolics Protect Type I Collagen by the Inhibition of Matrix Metalloproteinases, Collagenase, and Collagen Cross-linking

Huifang Li[1,2†], Tithi Roy[3†], Samuel T. Boateng[3], Hao He[1,2], Chang Liu[2], Weixi Liu[2], Dongli Li[1], Panpan Wu[1], Navindra P. Seeram[2], Jean Christopher Chamcheu[3*], and Hang Ma[1,2*]

[1] School of Biotechnology and Health Sciences, Wuyi University; International Healthcare Innovation Institute (Jiangmen), Jiangmen 529020, China

[2] Bioactive Botanical Research Laboratory, Department of Biomedical and Pharmaceutical Sciences, College of Pharmacy, University of Rhode Island, Kingston, RI 02881, USA

[3] School of Basic Pharmaceutical and Toxicological Sciences, College of Pharmacy, University of Louisiana at Monroe, Monroe, LA 71209, USA

† These authors contributed equally to this work.

* Correspondence: chamcheu@ulm.edu; Tel: +1318-342-6820 (J.C.C.); hang_ma@uri.edu; Tel.: +1-401-874-2711 (H.M.)

LIST OF CONTENTS

Figure S1. The cell viability at four (4) different concentration against A431 and NTERT1 for Maplifa®, Pomella®, GA, PA and their combinations.

Figure S2. Total ion chromatograms of Pomella® in liquid chromatography coupled with time-of-flight tandem mass spectrometry (LC-TOF-MS/MS)……………………………………………..…………..…3

Figure S3. Total ion chromatogram of Maplifa® in ultra-fast liquid chromatography coupled with electrospray ionization/time-of-flight mass spectrometry (UFLC-ESI-TOF-MS/MS)………………………………………………………………………………………………………..4

Table S1. Characterization of phenolic compounds in Pomella® by LC-TOF-MS/MS……………………………………………………………………………………………………....6

Table S2. Characterization of phenolic compounds in a methanolic extract red maple leaves and Maplifa® by LC-ESI-TOF-MS/MS…………………………………..………………………………………..12

Figure S1. The cell viability of Maplifa®, Pomella®, GA and PA at 12.5, 25, 50 and 100 µg/ml or µM in A431(A) and NTERT1(B).

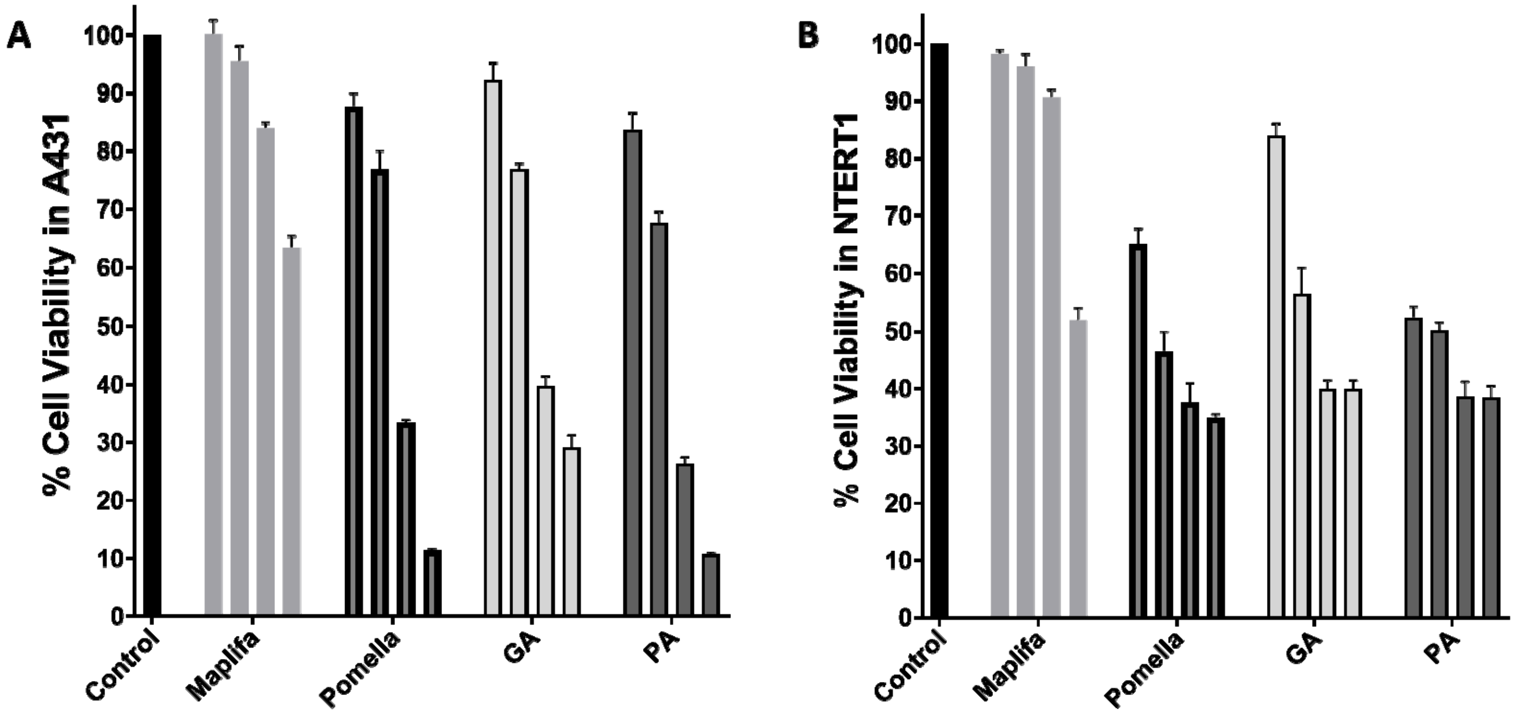

Figure S2. Total ion chromatograms of Pomella® in liquid chromatography coupled with time-of-flight tandem mass spectrometry (LC-TOF-MS/MS).

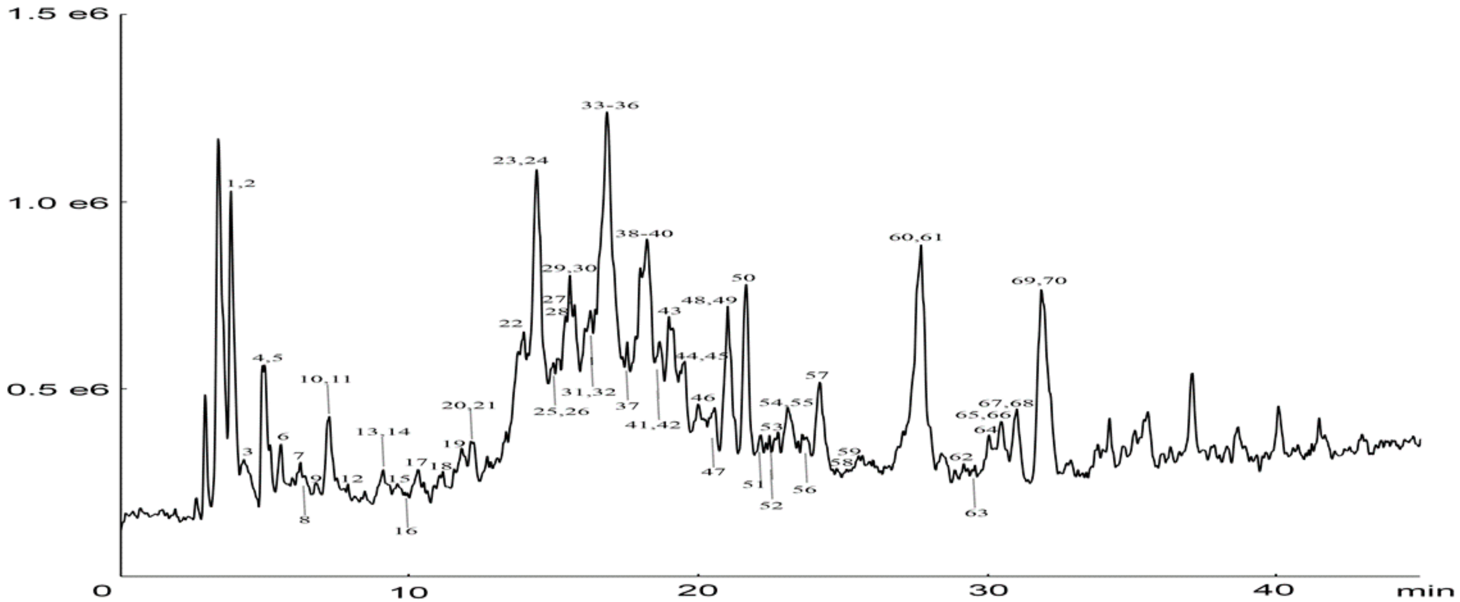

Figure S3. Total ion chromatogram of Maplifa® in ultra-fast liquid chromatography coupled with electrospray ionization/time-of-flight mass spectrometry.

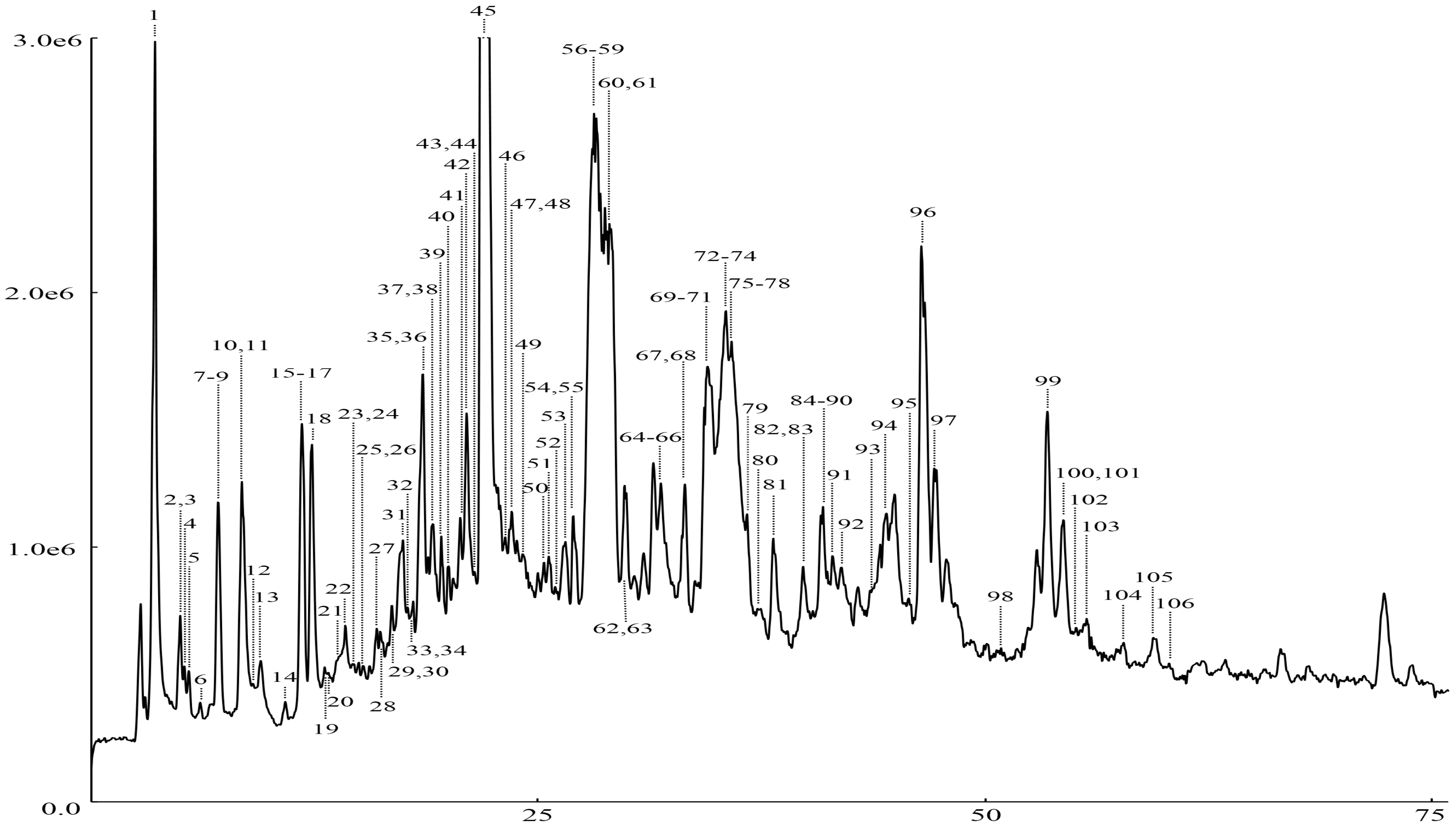

Table S1. Characterization of phenolic compounds in Pomella® by LC-TOF-MS/MS.

No	t_R (min)	Molecular formula	HR-ESI(-)-MS $[M-H]^-$ *m/z* exptl. (theor.)	Error (ppm)	Major MS/MS ions	Identification
1	3.80	$C_{20}H_{18}O_{14}$	481.0631 (481.0623)	1.4983	301, 275, 257, 229	HHDP-hexoside
2	3.92	$C_{13}H_{16}O_{10}$	331.0678 (331.0670)	2.2032	271, 211, 169, 125	galloyl-hexoside
3	4.40	$C_{20}H_{18}O_{14}$	481.0627 (481.0623)	0.6668	301, 275, 257, 229	HHDP-hexoside
4	4.88	$C_{20}H_{18}O_{14}$	481.0624 (481.0623)	0.0431	301, 275, 257, 229	HHDP-hexoside
5	4.99	$C_{13}H_{16}O_{10}$	331.0680 (331.0670)	2.8073	271, 211, 169, 125	galloyl-hexoside
6	5.47	$C_{13}H_{16}O_{10}$	331.0675 (331.0670)	1.2970	271, 211, 169, 125	galloyl-hexoside
7	6.17	$C_{13}H_{16}O_{10}$	331.0678 (331.0670)	2.2032	271, 211, 169, 125	galloyl-hexoside
8	6.36	$C_{27}H_{22}O_{19}$	649.0684 (649.0682)	0.2271	497, 301, 275, 229, 195	galloyl-HHDP-glucuronide

9	6.82	$C_{27}H_{22}O_{18}$	633.0732 (633.0733)	-0.2178	481, 463, 421, 301, 275, 257, 249, 229	galloyl-HHDP-hexoside
10	7.15	$C_{7}H_{6}O_{5}$	169.0155 (169.0142)	7.4136	125	gallic acid
11	7.20	$C_{13}H_{16}O_{10}$	331.0676 (331.0670)	1.5991	271, 211, 169, 125	galloyl-hexoside
12	7.74	$C_{34}H_{22}O_{22}$	781.0524 (781.0529)	-0.7637	721, 601, 575, 449, 393, 301, 300, 299, 271, 245, 229	gallagyl-hexoside
13	9.09	$C_{48}H_{28}O_{30}$	1083.0579 (1083.0592)	-1.2592	1065, 807, 781, 763, 721, 601, 575, 549, 341, 301, 300, 299, 275, 273, 271	HHDP-gallagyl-hexoside*
14	9.10	$C_{27}H_{22}O_{18}$	633.0737 (633.0733)	0.5719	481, 463, 301, 275, 257, 249, 229	galloyl-HHDP-hexoside
15	9.60	$C_{34}H_{24}O_{22}$	783.0684 (783.0686)	-0.3148	481, 437, 419, 341, 329, 301, 299, 275, 257, 229	Bis-HHDP-hexoside
16	9.83	$C_{27}H_{22}O_{19}$	649.0684 (649.0682)	0.2271	497, 301, 284, 275, 229, 195	galloyl-HHDP-glucuronide
17	10.31	$C_{48}H_{28}O_{30}$	1083.0586 (1083.0592)	-0.6129	807, 781, 763, 721, 601, 575, 549, 341, 301, 300, 299, 275, 273, 271	HHDP-gallagyl-hexoside*
18	10.93	$C_{13}H_{16}O_{10}$	331.0675 (331.0670)	1.2970	169, 125	galloyl-hexoside
19	11.86	$C_{34}H_{24}O_{22}$	783.0680	-0.8257	481, 301, 275, 257, 229	Bis-HHDP-hexoside

			(783.0686)			
20	12.08	$C_{41}H_{26}O_{26}$	933.0622 (933.0639)	-1.8811	781, 763, 721, 601, 575, 549, 449, 393, 301, 300, 299, 271	galloyl-gallagyl-hexoside
21	12.21	$C_{15}H_{14}O_{7}$	305.0674 (305.0666)	2.7313	261, 221, 179, 167, 165, 137, 125, 109	gallocatechin/epigallo catechin
22	13.80	$C_{27}H_{22}O_{18}$	633.0737 (633.0733)	0.5719	481, 463, 421, 301, 275, 257, 249, 229	galloyl-HHDP-hexoside
23	14.25	$C_{21}H_{10}O_{13}$	469.0056 (469.0048)	1.5687	425, 407, 379, 300, 279, 251, 195	valoneic acid bilactone
24	14.39	$C_{48}H_{28}O_{30}$	1083.0581 (1083.0592)	-1.0746	781, 721, 601, 575, 549, 301, 300, 299, 275, 273, 271	HHDP-gallagyl-hexoside*
25	14.94	$C_{27}H_{22}O_{18}$	633.0737 (633.0733)	0.5719	481, 463, 421, 301, 275, 257, 249, 229	galloyl-HHDP-hexoside
26	15.02	$C_{20}H_{20}O_{14}$	483.0785 (483.0780)	0.9743	423, 405, 331, 313, 271, 241, 211, 169, 125	digalloyl-hexoside
27	15.34	$C_{20}H_{20}O_{14}$	483.0775 (483.0780)	-1.0956	423, 331, 313, 271, 211, 169, 125	digalloyl-hexoside
28	15.39	$C_{41}H_{28}O_{27}$	951.0735 (951.0745)	-1.0724	907, 783, 605, 481, 425, 301, 275, 257, 249, 229	HHDP-valoneoyl-hexoside
29	15.47	$C_{27}H_{22}O_{18}$	633.0736 (633.0733)	0.4139	481, 463, 421, 301, 275, 257, 249, 229	galloyl-HHDP-hexoside

30	15.83	$C_{34}H_{24}O_{22}$	783.0676 (783.0686)	-1.3365	481, 301, 275, 257, 229	Bis-HHDP-hexoside
31	16.15	$C_{20}H_{20}O_{14}$	483.0793 (483.0780)	2.6304	423, 331, 313, 271, 211, 169, 125	digalloyl-hexoside
32	16.44	$C_{20}H_{20}O_{14}$	483.0785 (483.0780)	0.9743	423, 331, 313, 271, 241, 211, 193, 169, 125	digalloyl-hexoside
33	16.57	$C_{27}H_{22}O_{18}$	633.0731 (633.0733)	-0.3758	481, 301, 275, 257, 249, 229	galloyl-HHDP-hexoside
34	16.79	$C_{48}H_{28}O_{30}$	1083.0587 (1083.0592)	-0.5206	781, 721, 601, 575, 549, 301, 300, 299, 275, 273, 271	HHDP-gallagyl-hexoside*
35	17.06	$C_{20}H_{20}O_{14}$	483.0785 (483.0780)	0.9743	331, 313, 271, 211, 169, 125	digalloyl-hexoside
36	17.15	$C_{34}H_{26}O_{22}$	785.0836 (785.0842)	-0.8873	615, 483, 463, 419, 331, 313, 301, 275, 257, 249, 229, 169, 125	digalloyl-HHDP-hexoside
37	17.53	$C_{20}H_{20}O_{14}$	483.0787 (483.0780)	1.3883	439, 331, 313, 287, 169, 125	digalloyl-hexoside
38	17.79	$C_{20}H_{20}O_{14}$	483.0785 (483.0780)	0.9743	331, 313, 271, 211, 169, 125	digalloyl-hexoside
39	18.02	$C_{27}H_{24}O_{18}$	635.0869 (635.0889)	-3.2877	483, 465, 423, 313, 295, 271, 235, 211, 193, 169, 125	trigalloyl-hexoside
40	18.34	$C_{27}H_{22}O_{18}$	633.0737	0.5719	481, 463, 421, 301, 275, 257, 249, 229	galloyl-HHDP-

			(633.0733)			hexoside
41	18.57	$C_8H_8O_5$	183.0313 (183.0298)	7.6650	168, 140, 124	methyl gallic acid
42	18.69	$C_{34}H_{26}O_{23}$	801.0772 (801.0792)	-2.5107	649, 499, 347, 301, 275, 229, 195	digalloyl-HHDP-glucuronide
43	19.04	$C_{41}H_{28}O_{26}$	935.0781 (935.0796)	-1.6098	917, 783, 633, 571, 481, 419, 329, 301, 275, 257, 249, 231, 169, 125	galloyl-bis-HHDP-hexoside
44	19.27	$C_{27}H_{22}O_{18}$	633.0730 (633.0733)	-0.5338	481, 463, 421, 301, 275, 257, 249, 229	galloyl-HHDP-hexoside
45	19.43	$C_{13}H_8O_8$	291.0152 (291.0146)	1.9209	247, 219, 191	Brevifolin carboxylic acid
46	19.92	$C_{27}H_{24}O_{18}$	635.0886 (635.0889)	-0.6109	483, 465, 423, 331, 313, 295, 271, 211, 169, 125	trigalloyl-hexoside
47	20.69	$C_{34}H_{26}O_{22}$	785.0839 (785.0842)	-0.5022	615, 483, 463, 419, 331, 313, 301, 275, 257, 249, 229, 169, 125	digalloyl-HHDP-hexoside
48	21.00	$C_{37}H_{28}O_{23}$	839.0926 (839.0948)	-2.6950	537, 519, 479, 461, 435, 301, 273, 257, 229	pomellatannin
49	21.01	$C_{27}H_{24}O_{18}$	635.0886 (635.0889)	-0.6109	483, 465, 313, 169, 125	trigalloyl-hexoside
50	21.49	$C_{27}H_{22}O_{18}$	633.0737 (633.0733)	-0.5338	481, 463, 421, 301, 275, 257, 249, 229	galloyl-HHDP-hexoside

51	22.20	$C_{27}H_{24}O_{18}$	635.0882 (635.0889)	-1.2408	483, 465, 313, 211, 169, 125	trigalloyl-hexoside
52	22.52	$C_{34}H_{26}O_{22}$	785.0802 (785.0842)	-5.2181	615, 483, 463, 419, 331, 313, 301, 275, 257, 249, 229, 169, 125	digalloyl-HHDP-hexoside
53	22.68	$C_{21}H_{10}O_{13}$	469.0051 (469.0048)	0.5026	425, 407, 301, 300, 299, 271, 243	valoneic acid bilactone
54	22.94	$C_{27}H_{24}O_{18}$	635.0881 (635.0889)	-1.3982	483, 465, 313, 295, 271, 211, 169, 125	trigalloyl-hexoside
55	23.14	$C_{20}H_{16}O_{13}$	463.0514 (481.0518)	-0.4144	301, 300, 257, 229	ellagic acid-hexoside
56	23.81	$C_{27}H_{24}O_{18}$	635.0893 (635.0889)	0.4912	483, 465, 423, 331, 313, 295, 271, 221, 211, 169, 125	trigalloyl-hexoside
57	24.25	$C_{20}H_{16}O_{13}$	463.0515 (481.0518)	-0.3144	301, 300, 257, 229	ellagic acid-hexoside
58	24.73	$C_{34}H_{26}O_{22}$	785.0863 (785.0842)	2.5517	615, 483, 463, 419, 331, 313, 301, 275, 257, 249, 229, 169, 125	digalloyl-HHDP-hexoside
59	25.62	$C_{27}H_{24}O_{18}$	635.0885 (635.0889)	-0.7684	483, 465, 423, 313, 295, 271, 211, 169, 125	trigalloyl-hexoside
60	27.68	$C_{41}H_{28}O_{27}$	951.0713 (951.0745)	-3.3855	933, 915, 897, 765, 631, 613, 463, 445, 301, 275, 257, 249, 229, 169	galloyl-HHDP-DHHDP-hexoside
61	29.16	$C_{19}H_{14}O_{12}$	433.0411	-0.3458	301, 300, 257, 229	ellagic acid pentoside

			(433.0412)			
62	29.52	$C_{34}H_{28}O_{22}$	787.0980 (787.0999)	-2.4733	635, 617, 483, 465, 447, 423, 313, 295, 249, 211, 169, 125	tetragalloyl-hexoside
63	30.45	$C_{19}H_{14}O_{12}$	433.0408 (433.0412)	-1.0386	301, 300, 257, 229	ellagic acid-pentoside
64	30.68	$C_{34}H_{26}O_{22}$	785.0827 (785.0842)	-2.0337	615, 483, 463, 419, 331, 313, 301, 275, 257, 249, 229, 169, 125	digalloyl-HHDP-hexoside
65	30.89	$C_{34}H_{28}O_{22}$	787.0971 (787.0999)	-3.6167	635, 617, 573, 465, 313, 295, 211, 169, 125	tetragalloyl-hexoside
66	31.05	$C_{20}H_{16}O_{12}$	447.0571 (447.0568)	0.4476	301, 300, 257, 229	ellagic acid-deoxyhexoside
67	31.82	$C_{14}H_{6}O_{8}$	300.9986 (300.9989)	-1.2986	284, 257, 245, 229, 201, 185	ellagic acid
68	32.23	$C_{34}H_{28}O_{22}$	787.0972 (787.0999)	-2.7467	617, 465, 449, 423, 313, 295, 169, 125	tetragalloyl-hexoside
69	33.97	$C_{41}H_{30}O_{26}$	937.0930 (937.0952)	-2.4067	785, 767, 635, 617, 597, 483, 465, 445, 419, 313, 301, 275, 257, 249, 229, 169, 125	trigalloyl-HHDP-hexoside
70	35.61	$C_{41}H_{32}O_{26}$	939.1076 (939.1109)	-3.5197	787, 769, 635, 617, 599, 465, 447, 431, 313, 295, 169, 125	pentagalloyl-hexoside

* punicalagin isomers

Table S2. Characterization of phenolic compounds in a methanol extract red maple leaves and Maplifa™ by LC-ESI-TOF-MS/MS

No	t_R (min)	Formula	HR-ESI(-)-MS $[M-H]^-$ (*m/z* exptl (theor))	Error (ppm)		Identification
1	3.48	$C_7H_{12}O_6$	191.0568 (191.0561)	3.602	173, 155, 137, 127, 109	quinic acid
2	4.85	$C_{13}H_{16}O_{10}$	331.0680 (331.0670)	2.807	313, 271, 211, 169, 125	galloyl hexoside
3	4.98	$C_{14}H_{16}O_{10}$	343.0687 (343.0670)	4.749	191, 169, 125	galloyl quinic acid
4	5.23	$C_{13}H_{16}O_9$	315.0729 (315.0721)	2.362	169, 125	maplexin A
5	5.49	$C_{13}H_{16}O_{10}$	331.0679 (331.0670)	2.505	271, 211, 169, 125	galloyl hexoside
6	6.17	$C_{13}H_{16}O_{10}$	331.0686 (331.0670)	4.62	271, 211, 169, 125	galloyl hexoside
7	6.9	$C_{13}H_{16}O_9$	315.0735 (315.0721)	4.266	169, 125	maplexin B
8	7.06	$C_{14}H_{16}O_{10}$	343.0686 (343.0670)	4.458	191, 169, 125	galloyl quinic acid
9	7.13	$C_7H_6O_5$	169.0145 (169.0142)	1.497	125	gallic acid

10	8.18	$C_{13}H_{16}O_{10}$	331.0684 (331.0670)	4.016	313, 241, 211, 169, 125	galloyl hexoside
11	8.44	$C_{14}H_{16}O_{10}$	343.0682 (343.0670)	3.292	191, 173, 169, 125	4-*O*-galloyl quinic acid
12	9.01	$C_{13}H_{16}O_{10}$	331.0687 (331.0670)	4.922	313, 241, 169, 125	galloyl hexoside
13	9.46	$C_{13}H_{16}O_{9}$	315.0729 (315.0721)	2.362	169, 125	galloyl deoxyhexoside
14	10.8	$C_{13}H_{16}O_{10}$	331.0682 (331.0670)	3.411	169, 125	galloyl hexoside
15	11.6	$C_{14}H_{14}O_{9}$	325.0577 (325.0565)	3.674	281, 173, 169, 125	galloyl shikimic acid
16	11.8	$C_{13}H_{16}O_{9}$	315.0725 (315.0721)	1.092	169, 125	ginnalin B
17	12.4	$C_{13}H_{16}O_{9}$	315.0733 (315.0721)	3.631	169, 125	ginnalin C
18	13.1	$C_{14}H_{18}O_{10}$	345.0843 (345.0827)	4.577	183, 168, 124	methylgalloyl hexoside
19	13.7	$C_{20}H_{20}O_{14}$	483.0808 (483.0780)	5.735	331, 313, 271, 211, 169, 125	digalloyl hexoside
20	14.6	$C_{20}H_{20}O$	483.0816 (483.0780)	7.392	331, 271, 211, 169, 125	digalloyl hexoside

		14				
21	14.6	$C_{14}H_{14}O_{9}$	325.0577 (325.0565)	3.674	173, 169, 125	galloyl shikimic acid
22	15.2	$C_{20}H_{20}O_{14}$	483.0810 (483.0780)	6.149	331, 313, 271, 211, 169, 125	digalloyl hexoside
23	16	$C_{21}H_{20}O_{14}$	495.0817 (495.0780)	7.414	343, 191, 169, 125	digalloyl quinic acid
24	16.2	$C_{14}H_{14}O_{9}$	325.0583 (325.0565)	5.519	173, 169, 125	galloyl shikimic acid
25	16.8	$C_{20}H_{20}O_{13}$	467.0842 (467.0831)	2.324	315, 297, 169, 125	maplexin C
26	16.9	$C_{20}H_{20}O_{14}$	483.0816 (483.0780)	7.392	465, 331, 313, 271, 211, 169, 125	digalloyl hexoside
27	17.4	$C_{21}H_{20}O_{14}$	495.0806 (495.0780)	5.192	343, 191, 173, 169, 125	digalloyl quinic acid
28	17.7	$C_{20}H_{20}O_{14}$	483.0812 (483.0780)	6.564	465, 331, 313, 271, 241, 169, 125	digalloyl hexoside
29	18	$C_{15}H_{14}O_{6}$	289.0722 (289.0717)	1.515	245, 205, 203	catechin
30	18	$C_{14}H_{10}O_{9}$	321.0259 (321.0252)	2.163	169, 125	digallate

31	18.4	$C_8H_8O_5$	183.0313 (183.0298)	7.655	168, 124	gallic acid methyl ester
32	18.6	$C_{20}H_{20}O_{13}$	467.0862 (467.0831)	6.606	315, 297, 169, 125	3,6-di-*O*-galloyl-1,5-anhydroglucitol
33	18.9	$C_{20}H_{20}O_{14}$	483.0813 (483.0780)	6.771	465, 331, 313, 169, 125	digalloyl hexoside
34	19.1	$C_{20}H_{20}O_{13}$	467.0865 (467.0831)	7.248	315, 169, 125	digalloyl deoxyhexoside
35	19.6	$C_{20}H_{20}O_{13}$	467.0869 (467.0831)	8.104	315, 297, 169, 125	maplexin D
36	19.9	$C_{21}H_{18}O_{13}$	477.0704 (477.0674)	6.153	325, 201, 173, 169, 125	digalloyl shikimic acid
37	20.4	$C_{20}H_{20}O_{14}$	483.0812 (483.0780)	6.564	331, 313, 169, 125	digalloyl hexoside
38	21	$C_{15}H_{14}O_6$	289.0724 (289.0717)	2.207	245, 205, 203	epicatechin
39	21.3	$C_{20}H_{20}O_{13}$	467.0868 (467.0831)	7.89	423, 315, 169, 152, 125	digalloyl deoxyhexoside
40	22	$C_{20}H_{20}O_{13}$	467.0856 (467.0831)	5.321	315, 297, 211, 169, 125	ginnalin A
41	22.9	$C_{27}H_{24}O_{17}$	619.0973 (619.0940)	5.212	467, 315, 297, 169, 125	trigalloyl deoxyhexoside

42	23.6	$C_{21}H_{22}O_{13}$	481.1002 (481.0987)	2.983	315, 169, 125	galloyl methylgalloyl deoxyhexoside
43	23.7	$C_{27}H_{24}O_{17}$	619.0975 (619.0940)	5.535	467, 315,169, 125	trigalloyl deoxyhexoside
44	24.4	$C_{27}H_{24}O_{17}$	619.0973 (619.0940)	5.212	467, 315, 297, 169, 125	trigalloyl deoxyhexoside
45	24.9	$C_{26}H_{28}O_{16}$	595.1333 (595.1304)	4.774	301, 300, 271, 255, 243, 227, 179, 151	quercetin hexosyl pentoside
46	25.7	$C_{28}H_{24}O_{16}$	615.1035 (615.0991)	7.058	463, 301, 300, 271, 255, 243, 227, 211, 179, 169, 151, 125	quercetin-3-*O*-galloyl hexoside
47	26	$C_9H_{10}O_5$	197.0460 (197.0455)	2.298	182, 167, 123	di-methyl gallic acid
48	26.5	$C_{28}H_{24}O_{16}$	615.1042 (615.0991)	8.196	463, 301, 300, 271, 255, 243, 227, 211, 179, 169, 151, 125	quercetin-3-*O*-galloyl hexoside
49	27	$C_{14}H_5O_8$	300.9996 (300.9989)	2.024	284, 257, 229, 201, 185, 173, 145,	ellagic acid
50	27.4	$C_{27}H_{30}O_{16}$	609.1496 (609.1461)	5.731	301, 300, 271, 255, 243, 151	quercetin-3-*O*-hexosyl deoxyhexoside
51	27.7	$C_9H_{10}O_5$	197.0461 (197.0455)	2.806	182, 167, 123	5-methyl gallic acid methyl ester
52	27.8	$C_{22}H_{18}O_{10}$	441.0857 (441.0827)	6.755	289, 245, 169, 125	galloyl (epi)catechin

53	28.1	$C_{27}H_{24}O_{17}$	619.0977 (619.0940)	5.858	467, 315, 297, 169, 125	trigalloyl deoxyhexoside
54	28.6	$C_{22}H_{18}O_{10}$	441.0858 (441.0827)	7.208	289, 245, 169, 125	galloyl (epi)catechin
55	29.2	$C_{28}H_{24}O_{16}$	615.1021(615.0991)	7.708	463, 313, 301, 273, 255, 245, 229, 211, 179, 169, 151, 125	quercetin galloyl hexoside
56	29.2	$C_{27}H_{24}O_{17}$	619.0975 (619.0940)	5.535	467, 315, 297, 169, 125	trigalloyl deoxyhexoside
57	29.9	$C_{27}H_{24}O_{17}$	619.0978 (619.0940)	3.727	467, 449, 315, 297, 169, 125	trigalloyl deoxyhexoside
58	30	$C_{28}H_{24}O_{16}$	615.1039(615.0991)	4.782	463, 313, 301, 273, 255, 245, 229, 211, 179, 169, 151, 125	quercetin galloyl hexoside
59	31.9	$C_{20}H_{18}O_{11}$	433.0808(433.0776)	7.307	301, 300, 271, 255, 243, 151	quercetin-3-*O*-pentoside
60	32.2	$C_{21}H_{20}O_{11}$	447.0956 (447.0932)	5.177	285, 284, 255, 227	kaempferol-3-*O*-hexoside
61	32.5	$C_{15}H_{12}O_{9}$	335.0430 (335.0408)	6.4	183, 168, 124	methyl digallate
62	33	$C_{34}H_{28}O_{21}$	771.1099 (771.1050)	6.313	619, 467, 449, 315, 297, 169, 125	tetragalloyl deoxyhexoside
63	33.5	$C_{29}H_{22}O$	593.0966 (593.0936)	4.924	441, 289, 271, 245, 203, 169, 125	digalloyl (epi)catechin

		14				
64	34.3	$C_{21}H_{20}O_{11}$	447.0953 (447.0932)	4.506	301, 300, 271, 255, 243, 227, 151	quercetin-3-*O*-deoxyhexoside
65	34.8	$C_{34}H_{28}O_{21}$	771.1108 (771.1050)	7.48	619, 467, 315, 297, 169, 125	tetragalloyl deoxyhexoside
66	35.2	$C_{28}H_{26}O_{17}$	633.1142 (633.1097)	7.071	467, 315, 297, 169, 125	digalloyl methylgalloyl deoxyhexoside
67	35.4	$C_{27}H_{22}O_{15}$	585.0909 (585.0885)	3.941	433, 301, 273, 255, 179, 151	quercetin galloyl pentoside
68	35.5	$C_{34}H_{28}O_{21}$	771.1101 (771.1050)	6.572	619, 467, 315, 297, 169, 125	tetragalloyl deoxyhexoside
69	35.9	$C_{28}H_{26}O_{17}$	633.1113 (633.1097)	2.49	467, 315, 297, 169, 125	digalloyl methylgalloyl deoxyhexoside
70	36	$C_{20}H_{18}O_{10}$	417.0843 (417.0827)	3.787	285, 284, 255, 227	kaempferol-3-*O*-pentoside
71	36.2	$C_{29}H_{22}O_{14}$	593.0960 (593.0936)	3.913	441, 423, 289, 271, 245, 203, 169, 125	digalloyl (epi)catechin
72	36.2	$C_{34}H_{28}O_{21}$	771.1096 (771.1050)	5.924	619, 467, 315, 297, 169, 125	tetragalloyl deoxyhexoside
73	36.7	$C_{28}H_{26}O_{17}$	633.1140 (633.1097)	6.755	467, 315, 297, 169, 125	digalloyl methylgalloyl deoxyhexoside

74	37.5	$C_{34}H_{28}O_{21}$	771.1085 (771.1050)	4.497	619, 467, 315, 297, 169, 125	tetragalloyl deoxyhexoside
75	38.6	$C_{34}H_{28}O_{21}$	771.1098 (771.1050)	6.183	619, 467, 315, 297, 169, 125	tetragalloyl deoxyhexoside
76	39.8	$C_{29}H_{22}O_{14}$	593.0967 (593.0936)	5.093	441, 289, 271, 245, 203, 169, 125	digalloyl (epi)catechin
77	40.9	$C_{21}H_{20}O_{10}$	431.1001 (431.0983)	4.011	285, 284, 255, 227	kaempferol-3-*O*-deoxyhexoside
78	40.9	$C_{29}H_{22}O_{14}$	593.0969 (593.0936)	5.43	441, 289, 271, 245, 203, 169, 125	digalloyl (epi)catechin
79	41	$C_{41}H_{32}O_{25}$	923.1163 (923.1159)	0.335	771, 619, 467, 315, 297, 169, 125	pentagalloyl deoxyhexoside
80	41.1	$C_{27}H_{22}O_{15}$	585.0934 (585.0885)	8.214	433, 301, 300, 271, 255, 179, 151	quercetin-3-*O*-galloyl pentoside
81	41.3	$C_{29}H_{22}O_{14}$	593.0967 (593.0936)	5.093	441, 289, 271, 245, 203, 169, 125	digalloyl (epi)catechin
82	42	$C_{41}H_{32}O_{25}$	923.1230 (923.1159)	7.593	771, 619, 467, 315, 297, 169, 125	pentagalloyl deoxyhexoside
83	42.4	$C_{22}H_{16}O_{13}$	487.0547 (487.0518)	5.924	335, 183, 168, 124	methyl trigallate
84	42.8	$C_{28}H_{24}O$	599.1055 (599.1042)	2.096	447, 315, 301, 300, 271, 255, 243, 227, 179,	quercetin-3-*O*-galloyl

		15			151	deoxyhexoside
85	43.4	$C_{35}H_{30}O_{21}$	785.1257 (785.1206)	6.391	633, 619, 467, 315, 297, 169, 125	trigalloyl methylgalloyl deoxyhexoside
86	44.3	$C_{16}H_{14}O_{9}$	349.0574 (349.0565)	2.562	197, 182, 167, 123	di-methyl digallate
87	46.5	$C_{28}H_{24}O_{15}$	599.1088 (599.1042)	7.604	447, 301, 255, 179, 151	quercetin galloyl deoxyhexoside
88	47.3	$C_{28}H_{24}O_{15}$	599.1078 (599.1042)	3.556	447, 301, 255, 179, 151	quercetin galloyl deoxyhexoside
89	53	$C_{35}H_{28}O_{19}$	751.1214 (751.1152)	8.251	599, 447, 301, 255, 179, 151	quercetin digalloyl deoxyhexoside
90	53.5	$C_{28}H_{24}O_{14}$	583.1144 (583.1093)	8.696	431, 297, 285, 255, 227	kaempferol galloyl deoxyhexoside
91	53.8	$C_{35}H_{28}O_{19}$	751.1208 (751.1152)	7.452	599, 447, 301, 255, 179, 151	quercetin digalloyl deoxyhexoside
92	54.5	$C_{28}H_{24}O_{14}$	583.1133 (583.1093)	6.809	431, 297, 285, 255, 227, 169, 125	kaempferol galloyl deoxyhexoside
93	55.1	$C_{35}H_{28}O_{19}$	751.1205 (751.1152)	7.052	599, 447, 301, 255, 179, 151	quercetin digalloyl deoxyhexoside
94	59.6	$C_{35}H_{28}O_{18}$	735.1258 (735.1202)	7.498	583, 431, 297, 285, 255, 227, 169, 125	kaempferol digalloyl deoxyhexoside
